# Genome Sequence of a Novel Multiple-Antibiotic-Resistant Member of the *Erysipelotrichaceae* Family Isolated from a Swine Manure Storage Pit

**DOI:** 10.1128/genomeA.00978-16

**Published:** 2016-09-22

**Authors:** Bradd J. Haley, Seon Woo Kim, Terence R. Whitehead

**Affiliations:** a Environmental Microbial and Food Safety Laboratory, Beltsville Agricultural Research Center, USDA, Agricultural Research Service, Beltsville, Maryland, USA; b National Center for Agricultural Utilization Research, Bioenergy Research Unit, USDA, Agricultural Research Service, Peoria, Illinois, USA

## Abstract

The swine gastrointestinal tract and stored swine manure may serve as reservoirs of antibiotic resistance genes, as well as sources of novel bacteria. Here, we report the draft genome sequence of a novel taxon in the *Erysipelotrichaceae* family, isolated from a swine manure storage pit that is resistant to multiple antibiotics.

## GENOME ANNOUNCEMENT

Subtherapeutic feeding of various antibiotics to domesticated food animals has been commonly practiced to promote better health and animal performance. This practice has come under intense scrutiny in the United States and worldwide due to the role it might have on the increased development of bacterial antibiotic resistance and potential impact on human health (1). Research in our laboratories has focused on the study of commensal microbial populations of swine feces and manure stored in underground pits in the Midwest United States, in order to better understand their role as potential reservoirs of antibiotic-resistant bacteria and genes (2–4). To further delineate the antibiotic-resistant populations of these ecosystems, strictly anaerobic bacterial strains were isolated on media containing tetracycline and/or erythromycin. One strain was isolated and found to be resistant to these and other antibiotics.

Genomic DNA from strain MTC7 was extracted from an overnight culture using a DNeasy blood and tissue kit (Qiagen, Valencia, CA, USA). The extracted DNA was then cleaned using a Genomic DNA Clean and Concentrator-10 kit (ZymoResearch, Irvine, CA, USA). For whole-genome shotgun sequencing, DNA libraries were constructed using the Nextera XT kit (Illumina, La Jolla, CA, USA) according to the manufacturer’s protocol. The libraries were then sequenced using the Illumina NextSeq500 platform (Illumina). Raw reads were demultiplexed and cleaned of sequencing adaptors using the bcl2fastq software and then further cleaned for quality; contaminants were removed using Trimmomatic version 0.35 (5). A draft genome of the organism was assembled using SPAdes version 3.6.2 (6) and annotated using the NCBI Prokaryotic Genome Annotation Pipeline (PGAP) version 3.1 (http://www.ncbi.nlm.nih.gov/genome/annotation_prok). Based on the results of the genome sequencing and assembly, the genome size is estimated to be approximately 2.5 Mb, containing an estimated 2,428 total genes. The G+C content is 37 mol%. Analysis of the 16S rRNA gene indicates that this strain is a novel genus within the family *Erysipelotrichaceae*, most closely related to type strains of *Eubacterium tortuosum*, *Faecalicoccus pleomorphus*, and *Eubacterium dolichum*, among several others. A comparison of the 16S rRNA gene against the nucleotide database hosted by NCBI identified three taxa with 99% similarity to that of MTC7. These are identified as an uncultured bacterium clone CR_52, *Erysipelotrichaceae bacterium* ErySL, and the uncultured *Erysipelotrichi bacterium* clone SL122.

The presence of genes conferring antibiotic resistance was evaluated by two methods. Using the ResFinder function on the Center for Genomic Epidemiology web server (http://www.genomicepidemiology.org), *aph(3′)-III*, *ant(6)-Ia, aadE* (aminoglycoside resistance), *lnu(B)* (lincosamide resistance), *erm(B)* (macrolide resistance), and *tet(M)* (tetracycline resistance) were identified. Similarly, *ermB*, *aph(3′)-III*, *ant(6)-Ia*, *lnuB, tetM*, *lsa(E)* (lincosamide and pleuromutilin resistance), *sat-4* (streptothricin resistance), and ANT(9)-Ia (aminoglycoside resistance) were identified using the Resistance Gene Identifier (RGI) program hosted by the Comprehensive Antibiotic Resistance Database (CARD) (7).

### Accession number(s).

The draft genome sequences for the MTC7 strain have been deposited in NCBI GenBank under accession number LVZN00000000.
